# Correlation of Shear-Wave Elastography and Apparent Diffusion Coefficient Values in Breast Cancer and Their Relationship with the Prognostic Factors

**DOI:** 10.3390/diagnostics12123021

**Published:** 2022-12-02

**Authors:** Sebnem Orguc, Çağdaş Rıza Açar

**Affiliations:** Department of Radiology, Manisa Celal Bayar University Medical School, 45030 Manisa, Turkey

**Keywords:** breast, elastography, ADC, diffusion, molecular subtype

## Abstract

Background: Diffusion-weighted imaging and elastography are widely accepted methods in the evaluation of breast masses, however, there is very limited data comparing the two methods. The apparent diffusion coefficient is a measure of the diffusion of water molecules obtained by diffusion-weighted imaging as a part of breast MRI. Breast elastography is an adjunct to conventional ultrasonography, which provides a noninvasive evaluation of the stiffness of the lesion. Theoretically, increased tissue density and stiffness are related to each other. The purpose of this study is to compare MRI ADC values of the breast masses with quantitative elastography based on ultrasound shear wave measurements and to investigate their possible relation with the prognostic factors and molecular subtypes. Methods: We retrospectively evaluated histopathologically proven 147 breast lesions. The molecular classification of malignant lesions was made according to the prognostic factors. Shear wave elastography was measured in kiloPascal (kPa) units which is a quantitative measure of tissue stiffness. DWI was obtained using a 1.5-T MRI system. Results: ADC values were strongly inversely correlated with elasticity (r = −0.662, *p* < 0.01) according to Pearson Correlation. In our study, the cut-off value of ADC was 1.00 × 10^−3^ cm^2^/s to achieve a sensitivity of 84.6% and specificity of 75.4%, and the cut-off value of elasticity was 105.5 kPa to achieve the sensitivity of 96.3% and specificity 76.9% to discriminate between the malignant and benign breast lesions. The status of prognostic factors was not correlated with the ADC values and elasticity. Conclusions: Elasticity and ADC values are correlated. Both cannot predict the status of prognostic factors and differentiate between molecular subtypes.

## 1. Introduction

Breast cancer is the most common malignancy in women worldwide. It is also one of the leading causes of cancer-related deaths [1]. There are four main molecular subtypes of breast cancer: luminal A, luminal B, human epidermal growth factor receptor 2 (HER2) positive, and triple-negative tumors [2]. This classification is based on the expression of estrogen receptor (ER), progesterone receptor (PR), HER2, and Ki-67 proliferation index. Each subtype has different clinical characteristics, prognosis, and response to treatment. Different imaging features of each subtype are also reported in the literature [3,4,5].

Mammography is the only method proven for breast cancer screening [6]. Because of the high density of the breast, especially in younger women, mammography has low sensitivity, and ultrasound becomes useful [7]. Ultrasonography is recommended for evaluating palpable breast masses and further characterization of breast masses detected with mammography [8]. All patients diagnosed with breast cancer also undergo breast ultrasound (US) examination during the staging process.

In neoplastic development, the cell density increases, a desmoplastic reaction occurs, and connective tissue production increases. As a result, malignant tissue becomes stiffer. Current US technology integrates advanced methods in adjunct to B-mode imaging. Elastography assesses the stiffness of the tumor and further improves the US characterization of breast masses. Using SWE (shear-wave elastography) in addition to B-mode ultrasound increases the diagnostic performance for breast lesions, compared with conventional B-mode ultrasound alone [9]. The stiffness of breast cancer which is determined by elastography is analogous to the firmness of the mass detected by clinical breast examination. Elastography is not a screening tool, but it provides additional information to B-mode ultrasonography.

There are two main types of elastography: strain and shear-wave elastography. Strain elastography is a technique that is based on the relative displacement of the tissue by the pressure which is generated via compression with a transducer. With strain elastography, quantitative measurement is not possible. Unlike strain elastography and clinical breast examination, shear-wave elastography is a quantitative method in the detection of stiffness. SWE using the acoustic radiation force induced by the ultrasound push pulse generated by the transducer provides quantitative elasticity parameters, as well as displaying a visual color overlay of elastic information in real-time [10]. The parameters of SWE are the minimum (Emin), maximum (Emax), and mean (Emean) elasticity. Between the SWE parameters, the mean stiffness (Emean) has been shown to be the most reliable measurement of elasticity [11].

Another method for the detection of breast malignancy is Magnetic Resonance Imaging (MRI). Indications of Breast MRI are staging of known cancer, screening for breast cancer in women at increased risk, and evaluation of response to neoadjuvant chemotherapy [12]. Breast MRI is a multiparametric technique which incorporates dynamic T1-weighted contrast-enhanced, T2-weighted, and diffusion-weighted imaging (DWI) sequences. The advantages of DWI over contrast-enhanced MRI are short scanning time and no need for contrast material [13].

Diffusion is the movement of molecules from a region of higher concentration to a region of lower concentration. Water contains abundant hydrogen nuclei, which can be detected by MRI scanners. The movement of water molecules is influenced by tissue microstructure and cell density.

On DWI, we use gradient pulses. Water molecules that do not move between gradient pulses refocus and generate high signals. Moving water molecules will cause dephasing, which results in a hypointense DWI signal. Therefore, on DWI, the areas of restricted diffusion appear bright, and the areas of free diffusion appear dark.

**Figure 1:**
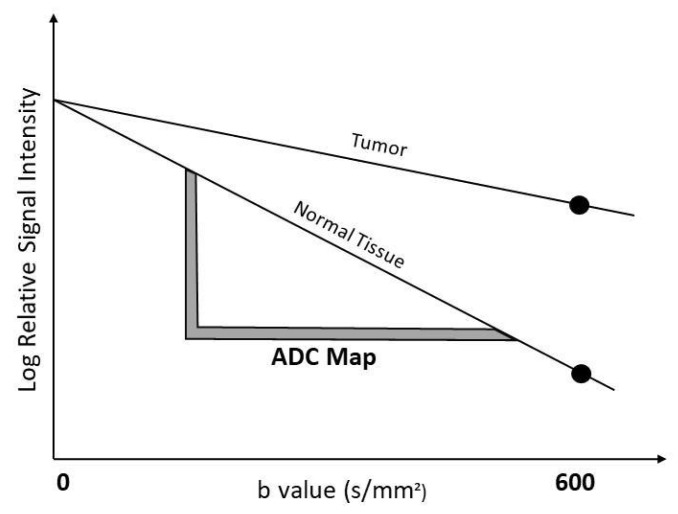
The graph illustrates b values versus the logarithm of signal intensity at diffusion-weighted imaging of normal tissue versus tumor. The signal of water molecules reduces with high b values, which causes a low ADC value.

The b-value is a factor that reflects the strength and timing of the gradients used to generate diffusion-weighted images (Figure 1). The apparent diffusion coefficient (ADC) is a measure of the amount of diffusion occurring in each voxel. ADC is calculated from an image with no diffusion weighting combined with one or more diffusion-weighted images. As a result, tumor cell density and microstructure are related to ADC. The higher the cell density and more complex microstructure, the lower the ADC value.

The relationship between tissue elasticity and cellularity–microstructure, and thus elastographic and ADC measurements and association of these features with the molecular subtypes, is not well established in breast lesions. Kapetas et al. evaluated the relationship between ARFI (Acoustic Radiation Force Impulse Elastography) and DWI in breast lesions [14]. Matsubayashi et al. evaluated the relationship between elastographic strain score and DWI according to the degree of fibrosis based on pathological examinations [15]. Baltacioglu et al. evaluated the relationship between SWE and DWI in fibroadenomas [16]. The association between prognostic factors, molecular subtypes, and elasticity, ADC values were investigated before, but results are discordant [17,18,19,20,21,22,23,24,25,26,27,28,29]. The purpose of this study was to compare the ADC values measured with DWI and shear wave properties quantified by US elastography in breast lesions and to investigate their possible relation with the prognostic factors and molecular subtypes.

## 2. Materials and Methods

### 2.1. Study Group

This retrospective study was approved by the Ethics Committee of Manisa Celal Bayar University School of Medicine (Approval Date: 15 January 2020, Approval No: 20478486-050.04.04). A total of 144 consecutive patients with 147 breast lesions referred for diagnosis and pre-treatment staging of breast cancer underwent both SWE and MRI within 2 weeks from March 2017 to February 2021 were included in the study. The inclusion criteria for breast lesions were as follows: Solid breast lesions or complex masses with <25% cystic component, no previous history of breast surgery, radiation or chemotherapy, pathological confirmation of breast cancer with image-guided core needle biopsy or surgery.

### 2.2. Elastography Examination Technique

We used the Toshiba Aplio 500 system (Toshiba Medical System Corporation, Tochigi, Japan), which exhibits the stiffness distribution of lesions both in KPa (range, 0–180 KPa) and in m/s (range, 0.5–8.0 m/s). All measurements were obtained by the same radiologist with more than 30 years of experience with breast US. US examination was performed with a 14L5 linear array hand hold transducer (frequency range: 5–15 MHz) with the patient in the supine position. B-mode images were obtained in two planes perpendicular to each other. Subsequently, SWE examinations of the target breast lesions were carried out by keeping the transducer in a vertical position and applying a generous amount of gel. Patients were instructed to hold their breath while the SWE images were obtained. During the US examination, a careful technique was carried out to obtain proper elastographic measurements without artifacts. Elastographic images were displayed in the elasticity mode with color coding. SWE map displayed the SWS distribution of the lesion corresponding to the degree of stiffness in ascending order of blue, green, yellow, and red. Areas that are not color-coded on elasticity images indicate that no shear wave is detected. The propagation mode was used to verify the reliability of the measurements. Nearly straight and regularly parallel contour lines confirm reliable data, whereas chaotic, irregular, and distorted contour lines reflect unreliable measurements. The SWE examination was repeated until acceptable parallel contour lines were obtained. The lines of propagation are wider apart in stiff tissues, and the interval decreases in soft tissues.

**Figure 2:**
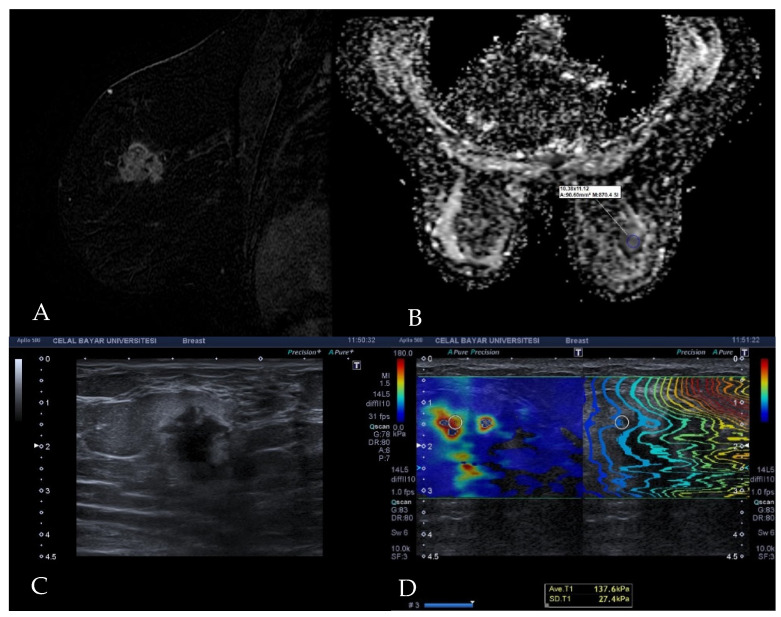
Images of invasive ductal carcinoma in a 36-year-old female patient. (**A**) Contrast-enhanced sagittal T1w image demonstrates a mass with irregular shape and contours and heterogeneous enhancement. (**B**) The lesion has a low signal on the ADC map with the value of 0.77 × 10^−3^ cm^2^/s. (**C**) B-mode ultrasonography shows a hypoechoic mass with angular margins. (**D**) Shear wave elastography reveals the mass is stiff with an elasticity value of 137.6 kPa.

A standard ROI (region of interest) was placed on areas with parallel contours on the propagation map at the stiffest part of the target lesion, as indicated by the color mapping, and thus the shear wave property of the breast cancer was quantified in KPa units (Figure 2). All images and measurements were recorded in the picture archive communication system (PACS).

### 2.3. Diffusion MRI Examination Technique

Diffusion-weighted breast imaging was conducted with a 1.5 Tesla MRI machine (Signa HDx; General Electric, Madison, WI, USA) and bilateral 8-channel high-density breast coil as a part of routine dynamic post-contrast breast MRI examination. The DWI sequence was obtained in the axial plane with b-values of 0 s/mm^2^ and 600 s/mm^2^ prior to contrast administration. The parameters of DWI were as follows: (a) sequence (echo planar imaging); (b) Repetition time (TR)/time to echo (TE): 900 ms/88.9 ms (c) Field of view (FOV: 36–40 cm); (d) matrix: 192 × 192; (e) slice thickness/interval: 5 mm/1 mm; (f): NEX (square root of the number of excitations): 16; (g) rBW (receiver bandwidth): 250 kHz. The total imaging time of the DWI sequence was 261 s. The images were transferred to the workstation (Advantage Windows 4.6; General Electric, Madison, WI, USA) to generate a black-and-white apparent diffusion coefficient map. The ADC value of the target breast lesion was measured on a single section containing the largest available tumor area, with an ROI 10–100 mm^2^ in size. The ROI was placed manually on the solid part of the tumor, corresponding to enhancing areas, taking care to omit normal breast tissue as well as areas of necrosis and hemorrhage (Figure 2).

### 2.4. Histopathological Analysis

We investigated histopathological results from Tru-cut biopsies or excisional biopsy materials to make an immunohistochemical classification of the lesions. ER, PR, HER2 expressions, and Ki-67 index were evaluated. ER and PR were accepted as positive if equal or more than 1%. HER2 expression was scored between 0 and 3. Scores of 0 and 1 were accepted as HER2 negative, a score of 3 was accepted as positive. In the case of a score of 2, HER2 gene amplification was evaluated by fluorescent in situ hybridization (FISH) test to determine the positivity or negativity of HER2. Lesions with Ki-67 staining of ≥14% were categorized as high-expression, and lesions with Ki-67 staining of <14% were categorized as low-expression.

Tumors with ER positive or PR positive/HER2 negative with low Ki-67 expression were classified as Luminal A. Tumors with ER positive or PR positive/HER2 positive or HER2 negative with high Ki-67 expression were classified as Luminal B. Tumors with ER negative and PR negative/HER2 positive were classified as HER2 positive subtype. Tumors with ER negative, PR negative, and HER2 negative were classified as Triple-negative subtype (Table 1).

### 2.5. Statistical Analysis

Statistical analysis was performed with SPSS version 23 (IBM Corp, New York, NY, USA). Correlation between ADC values and elasticity was calculated using Pearson’s correlation coefficients (PCC). Receiver operating characteristics (ROC) curve analysis was used to evaluate the accuracy of ADC value and elasticity to discriminate between benign and malignant lesions based on histopathological results.

The normality of the ADC values and elasticity distribution was assessed using the Shapiro-Wilk test. To identify statistically significant differences between elasticity and ADC values among lesion groups, we used Mann-Whitney *U*-test and the Kruskal-Wallis test. In order to examine whether the ADC values and elasticity can provide prognostic information, the difference in ADC values and elasticity of the different prognostic groups was analyzed. In cases in which the prognostic groups were classified as two categories, the Mann-Whitney *U*-test was used, and in cases in which the prognostic groups were classified as more than three categories, as seen in molecular subgroups, the Kruskal-Wallis test was used. A two-tailed *p* value of <0.05 was accepted as significant.

## 3. Results

All patients were female. The mean age of the patients was 52.75 years, within a range of 25–82 years. The median diameter of the breast tumors was 32.50 mm.

134 of 147 detected lesions were malignant, and 13 were benign. Benign lesions included fibrocystic changes (*n* = 4; 30.76%), inflammatory changes (*n* = 1), ductal hyperplasia (*n* = 1), fat necrosis (*n* = 1), apocrine metaplasia (*n* = 1), phyllodes tumor (*n* = 1), radial scar (*n* = 1), fibroadenoma (*n* = 1), sclerosing adenosis (*n* = 1), fibrotic changes (*n* = 1) (Table 2).

134 malignant lesions included invasive ductal carcinoma (*n* = 115; 85.82%), invasive lobular carcinoma (*n* = 13; 9.7%), ductal carcinoma in situ (*n* = 3; 2.23%), malignant epithelial tumor (*n* = 1; 0.74%), mucinous carcinoma (*n* = 1; 0.74%) and malignant phyllodes tumor (*n* = 1; 0.74%) (Table 2).

Malignant phyllodes tumors are not included in the molecular classification. Therefore 133 malignant lesions were classified based on molecular status. Estrogen receptor positivity was detected in 108 (81.2%) lesions. 82 (61.65%) lesions tested positive for progesterone receptor, and 35 (36.31%) lesions tested positive for HER2 protein. A high Ki-67 index was seen in 92 (69.17%) lesions (Table 3).

Among 133 malignant lesions, 37 (27.61%) of them were Luminal A, 69 (51.49%) of them were Luminal B, 12 (8.95%) of them were HER2 positive subtype and 15 (11.19%) were triple-negative subtype (Table 4).

The median ADC value of all lesions was 0.93 × 10^−3^ cm^2^/s. The median elasticity of all lesions was 134 kPa. After categorizing the lesions as benign and malignant, the median ADC value of malignant lesions was 0.92 × 10^−3^ cm^2^/s, and the median ADC value of benign lesions was 1.60 × 10^−3^ cm^2^/s. ADC values of malignant lesions were significantly lower than benign lesions (*p* < 0.01). The median elasticity of malignant lesions was 135 kPa, and the median elasticity of benign lesions was 27 kPa. The elasticity of malignant lesions was significantly higher than benign lesions (*p* < 0.01).

**Figure 3:**
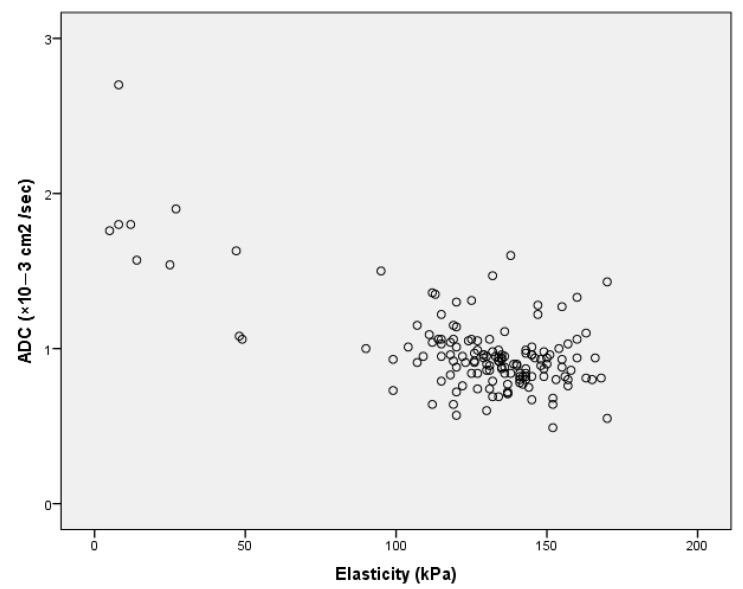
The graph shows the inverse linear correlation between elasticity and apparent diffusion coefficient (ADC) values.

ADC values were strongly inversely correlated with elasticity (r = −0.662, *p* < 0.01) according to Pearson Correlation (Figure 3). When the cut-off value of ADC was accepted as 1.00 × 10^−3^ cm^2^/s, we achieved a sensitivity of 84.6% and specificity of 75.4% in our study. If the cut-off value was accepted as 1.48 × 10^−3^ cm^2^/s, we achieved a sensitivity of %100 and a specificity of 76.9%. The cut-off value of elasticity was determined as 105.5 kPa to achieve a sensitivity of 96.3% and specificity of 76.9% for discriminating malignant and benign breast lesions.

**Figure 4:**
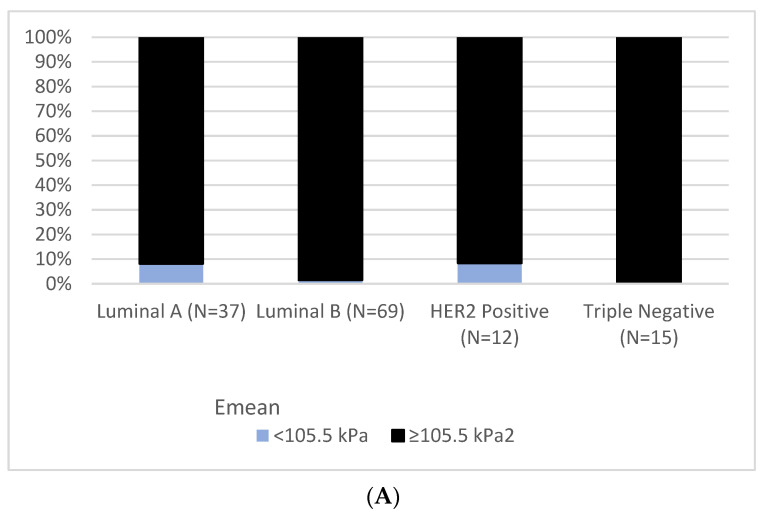

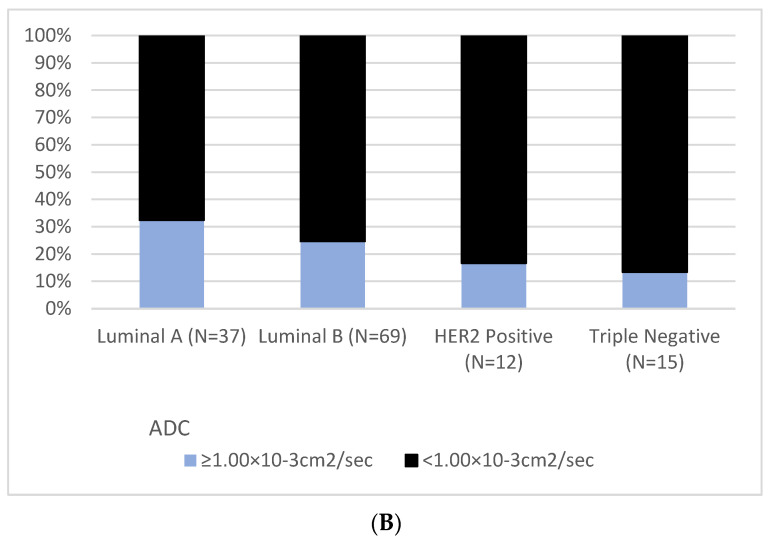
Tumors that were above and below the cut-off for (**A**) elasticity and (**B**) ADC value, respectively, according to the molecular subtype. Overall, there were more false negative cases in DWI than in elastography. Lesions above ADC cut-off value were higher in Luminal subtypes.

According to the molecular subtypes, tumors that were below and above the cut-off for elasticity and ADC values according to the molecular subtypes are illustrated in Figure 4.

ER, positivity, PR positivity, HER2 expression, and Ki−67 index were not significantly correlated with ADC values and elasticity (Table 3). ADC values and elasticity could not differentiate molecular subtypes of breast malignancies in our study (Table 4).

Among benign and malignant lesions, morphological features were not significantly associated with ADC values and elasticity (Table 5). Moreover, there was no significant correlation between lesion morphology and molecular subtypes (Table 4).

## 4. Discussion

Both US and DWI are non-invasive, radiation-free, and effective imaging methods that are widely used in the characterization and differential diagnosis of breast lesions. DWI quantifies the random motion of molecules, and SWE measures the elasticity in living tissues.

The application of US elastography in breast lesions has several advantages in comparison to other anatomical sites. Since the breast is a superficial organ, free of interfering factors such as vascular pulsations, SWE improves diagnostic confidence and has gained popularity among breast radiologists.

Our study evaluated the association between ADC values, elasticity, and molecular subtypes of breast tumors. A strong inverse correlation between elasticity and ADC values in breast lesions was found (*p* < 0.01). To the best of our knowledge, there is highly limited data regarding the correlation between elasticity determined by shear wave US examination and ADC value measured by diffusion-weighted MRI of breast lesions. Kapetas et al. evaluated 65 breast lesions and reported a significant correlation between quantitative findings of ARFI and DWI in breast lesions [14]. Matsubayashi et al. reported a significant correlation between relative elastographic strain score and MRI diffusion with the degree of fibrosis in breast lesions based on pathological examination [15]. Baltacioglu et al. evaluated 50 fibroadenomas in their study and reported a strong inverse correlation between elastographic measurements and ADC values [16].

We found the median ADC value of malignant lesions was 0.92 × 10^−3^ cm^2^/s. In the literature range of mean ADC value of malignant lesions reported varying from 0.89 ± 0.28 × 10^−3^ cm^2^/s to 1.00 × 10^−3^ cm^2^/s [27,30,31]. Our median ADC value of benign lesions was 1.60^−3^ cm^2^/s. In the literature range of mean ADC value of benign lesions reported varying from 1.1 ± 0.34 × 10^−3^ cm^2^/s to 1.57 ± 0.23 × 10^−3^ cm^2^ [27,30,31,32]. Different ADC cut-off values were reported to discriminate malignant from benign breast lesions in the literature. Surov et al., in their meta-analysis, which is based on 13.847 lesions, recommended a cut-off value of ADC was 1.00 × 10^−3^ cm^2^/s [28]. In our study, we achieved a sensitivity of 84.6%, and a specificity of 75.4% with a cut-off value of ADC was 1.00 × 10^−3^ cm^2^/s. The cut-off values reported for distinguishing benign and malignant breast lesions in other studies were 1.30 × 10^−3^ cm^2^/s by Guo et al. [31], 1.1 × 10^−3^ cm^2^/s by Azab et al. [33], 1.08 × 10^−3^ cm^2^/s by Akın et al. [34].

In our study, the median elasticity of malignant lesions was 135 kPa. In the literature, mean elasticity was reported between 130.7–179 kPa for malignant lesions in different studies [18,35,36,37,38]. For benign lesions, our median elasticity was 27 kPa. In the literature, mean elasticity was reported between 24.8–55.05 kPa [18,35,36,37,38]. 105.5 kPa cut-off value of elasticity resulted in a sensitivity of 96.3% and specificity of 76.9% in discriminating malignant and benign breast lesions in our study. In literature, the cut-off value is reported between 42.5–89.1 kPa [37,38,39,40]. 

There were two lesions that showed false positivity. Radial scar and fat necrosis, despite being benign lesions, had lower ADC and higher elasticity values than the cut-off value. For radial scar, the ADC value was 0.92 × 10^−3^ cm^2^/s, and elasticity was 126 kPa. Fat necrosis had an ADC value of 0.8 × 10^−3^ cm^2^/s and an elasticity of 43 kPa. Both lesions are known to be troublesome lesions for radiologists due to imaging features mimicking malignancy. The radial scar is characterized by a central area resembling scar tissue, ducts showing obliterative mastopathy, and surrounding elastic fibers [41]. Fat necrosis is characterized by sterile inflammation. At late stages, inflammatory components are replaced by fibrosis which leads to scar formation [42]. Pathological features explain the low ADC values and high elasticity in these two lesions. Evans et al. reported two cases of radial scar and fat necrosis, which had elasticity above the cut-off value [43].

Breast lesions are also classified according to their morphology as mass and non-mass enhancement (NME). A mass is a three-dimensional lesion that occupies a space in the breast, and which has clear boundaries. NME is an enhancing area without clear detectable boundaries. The contrary to mass lesions, NME lesions are not well defined. NME lesions usually contain normal fibroglandular tissue interspersed. Therefore, the measurement of ADC values of NME lesions inevitably includes some normal fibroglandular tissue also. In our study, among malignant lesions, masses had lower ADC values and slightly higher elasticity than NME, although it was not significant (Table 5). Among benign lesions, NME lesions had lower ADC values and higher elasticity, which was not significant also (Table 5). Cheng et al., in their study of 188 lesions, reported that there was a significant ADC value difference between mass and NME in the benign lesions. In malignant lesions, masses had lower ADC values, but the difference was not significant [44].

Breast imaging modalities improved very fast in recent years. At the beginning of the breast imaging journey about a century ago, the radiologic detection of lesions in the breast was accepted as a great success; however, today, we are investigating the possibility of differentiating molecular subtypes of breast cancer by imaging. Different imaging features of molecular subtypes have been reported several times in literature, however, with inconsistent findings across studies.

The stiffness of a tumor is determined by many factors, including cellularity, fibrosis, and necrosis. While Guo et al. evaluated tumor cellularity by pathological specimens and ADC values and found a significant correlation between them, Yoshikawa et al. used the same methods and found no significant correlation [31,45]. Squillaci et al. [46] investigated renal tumors, and Sugahara et al. [47] investigated gliomas in the same aspect and found no significant correlation.

These factors, which affect the stiffness of the tumor thought to be correlated with the prognostic factors. Ki-67 is a human nuclear protein used in routine pathology as a proliferation marker that is associated with cell proliferation [29]. ER is a predictive biomarker that can foresee patients likely to benefit from hormonal therapy. Patients with lesions showing ER and/ or PR positivity have lower risks of mortality when compared with patients with lesions which lack hormone receptor positivity [48]. While some studies said ER negativity inhibits the angiogenic pathway and causes a decrease in perfusion, some suggested that ER directly affects cellularity [24,49,50]. The relationship between ER positivity and high cellularity is well established [51,52]. PR negativity is related to a more aggressive subtype of ER + breast cancer [53]. PR was assumed to be a biomarker of an active ER pathway, but now it is accepted that PR has a direct functional role in the progression of tumors [54]. Overexpression of HER2 is another marker for poor prognosis of breast cancer [55]. HER2 gene encodes a transmembrane tyrosine kinase receptor which has a role in the regulation of cell growth, differentiation, and survival [56]. Abnormality in HER2 can cause uncontrolled cell proliferation [57].

Another factor that must be considered, which affects the tumor stiffness and ADC values, is the extracellular matrix. Matsubayashi et al. evaluated fibrotic changes in extracellular matrix and elasticity and reported the correlation between them [15]. The correlation between molecular subtypes and fibrotic changes also has contradictory and nondefinitive results reported in the literature [58,59,60,61,62].

The necrotic component is another factor that affects the stiffness and ADC values. HER2-positive and triple-negative tumors are known to have more necrotic components, which leads to low elasticity and high ADC values [63,64].

Therefore, the factors which effect ADC values and elasticity is complex and affected by many factors.

In our study, ADC values and elasticity showed no correlation with prognostic factors (ER, PR, HER2, Ki-67 index), histopathological types, and molecular subtypes. Their association with the ADC values and elasticity was investigated many times, but the results are discordant. The results of selected studies and the method they used are summarized in Table 6.

The most reasonable explanation for this situation is the usage of different methodologies in studies. The method for classifying molecular subtypes differentiated among studies. Some studies used different cut-off values to determine ER, and PR positivity, and some studies used the Method of Quick (Allred) Score (QS). Moreover, different cut-off values of the High or Low Ki-67 proliferation index are used. While some studies accepted a Score of 2 of HER2 as positive, some studies made further evaluation and evaluated HER2 amplification by fluorescent in situ hybridization to determine positivity. In the evaluation of elastographic examination, interobserver variability, operator dependency, technique difference, and in the evaluation of diffusion images difference in used b values, magnetic field strength, size of the ROIs, interobserver variability were the factors that affected the results [17,19,20,21,22,23,24,25,26,27,29,65,66].

While most studies that evaluated elasticity reported a correlation between lesion size and elasticity, none of the studies reported a correlation between lesion size and ADC values. Lesion size correlated neither with elasticity nor with ADC value in our study. Further prospective studies with uniform methodology will clarify the relation between elasticity, DWI, lesion size, and morphology.

### Study Limitations

The retrospective design of the study is one of the limitations of our study. Misregistration of the two measurements is another possible limitation. Manually drawn ROIs on the ADC map may show variations and may not correspond exactly to the ROI, which is also manually drawn on the elastographic image. Fusion imaging technologies may ensure more accurate registration of the ROIs used in two modalities. The ratio of malignant to benign cases was asymmetrical, with only 13 benign lesions. We also did not make interobserver and intraobserver measurements of the lesions. However, previous studies demonstrated the high reproducibility of the elastography measurements [67].

## 5. Conclusions

The diffusion property of breast cancer measured by DWI as ADC values inversely correlate with the stiffness of the tumor as measured by shear wave elastography. The ADC value decreases as the shear wave elasticity increases in a linear fashion in our study population. We also found no significant difference in diffusion and elastographic properties among molecular subtypes and prognostic factors of breast cancer.

## Figures and Tables

**Table 1:** Molecular subtypes and prognostic factors.

	Estrogen Receptor	Progesterone Receptor	HER2	Ki-67 Index
Luminal A	+	+/−	−	<14%
Luminal B				
Luminal B (HER2 negative)	+	+/−	−	≥14%
Luminal B (HER2 positive)	+	+/−	+	
HER2 + Subtype	−	−	+	
Triple-negative Subtype	−	−	−	

HER2, Human Epidermal Growth Factor 2; Ki-67, Nuclear protein Ki-67. Luminal A, tumors with ER positive or PR positive/HER2 negative with low Ki-67 expression; Luminal B, tumors with ER positive or PR positive/HER2 positive or HER2 negative with high Ki-67 expression; HER2 + subtype, tumors with ER negative and PR negative/HER2 positive; Triple-negative subtype, tumors with ER negative, PR negative and HER2 negative.

**Table 2:** Breast lesions are classified according to histopathological findings.

Histopathological Type of Malignant Lesions	Number of Lesions
	*n*
Malignant	**134**
Invasive ductal carcinoma	115
Invasive lobular carcinoma	13
Ductal carcinoma in situ	3
Malignant epithelial tumor	1
Mucinous carcinoma	1
Malignant phyllodes tumor	1
Benign	**13**
Fibrocystic changes	4
Inflammatory changes	1
Ductal hyperplasia	1
Fat necrosis	1
Apocrine metaplasia	1
Phyllodes tumor	1
Radial scar	1
Fibroadenoma	1
Sclerosing adenosis	1
Fibrotic changes	1

**Table 3:** Elasticity and ADC values of malignant lesions are classified according to the prognostic factor status of the tumor.

Characteristics	Number of Lesions *n* (%)	ADC (Median [IQR], ×10^−3^ cm^2^/s)	Elasticity (Median [IQR], kPA)	*p* Value
Estrogen				
*Positive*	108 (81.2%)	0.84 (0.30)	135.00 (24.00)	ADC → 0.323 Elasticity → 0.530
*Negative*	25 (18.8%)	0.94 (0.09)	136.00 (24.50)	
Progesterone				
*Positive*	82 (61.65%)	0.88 (0.19)	135.33 (28.00)	ADC → 0.211 Elasticity → 0.422
*Negative*	41 (38.35%)	0.94 (0.15)	136.00 (23.00)	
HER2				
*Positive*	35 (26.31%)	0.95 (0.17)	134.00 (29.00)	ADC → 0.051 Elasticity → 0.812
*Negative*	98 (73.69%)	0.88 (0.25)	130.00 (19.00)	
Ki-67 Proliferation Index				
*High*	92 (69.17%)	0.89 (0.18)	137.00 (22.50)	ADC → 0.638 Elasticity → 0.240
*Low*	41 (30.83%)	0.91 (0.25)	131.00 (22.50)	

ADC, apparent diffusion coefficient; IQR, inter quartile range; kPA, kilopascal; HER2, Human Epidermal Growth Factor 2; Ki-67, Nuclear protein Ki-67.

**Table 4:** Tumor size, lesion morphology, elasticity, and ADC values of molecular subtypes.

Characteristics	Number of Lesions *n* (%)	ADC (Median ± [IQR], ×10^−3^ cm^2^/s)	Elasticity (Median ± [IQR], kPA)	Tumor Size (Median ± [IQR], mm)	Lesion Morphology	
					Mass *n* (%)	Non-Mass *n* (%)
Malignant Lesions	134 (91.15%)	0.92 (0.18)	135.00 (24.45)	25.00 (22.00)	117 (87.2%)	17 (12.8%)
*Luminal A*	37 (27.61%)	0.90 (0.29)	131.00 (22.50)	20.00 (17.00)	33 (89.2%)	4 (10.8%)
*Luminal B*	69 (51.49%)	0.91 (0.19)	135.00 (23.00)	27.00 (24.50)	59 (85.5%)	10 (14.5%)
*HER2 positive*	12 (8.95%)	0.93 (0.16)	142.00 (20.00)	21.50 (19.00)	10 (83.3%)	2 (16.7%)
*Triple-negative*	15 (11.19%)	0.95 (0.09)	136.00 (35.00)	32.00 (17.00)	14 (93.3%)	1 (6.7%)
Benign Lesions	13 (8.84%)	1.60 (0.55)	27.00 (105.00)	20.00 (39.50)	6 (46.1%)	7 (53.8%)
All lesions	147 (100%)	0.93 (0.23)	134.00 (25.75)	34.50 (22.00)	123(83.7%)	24(16.3%)

ADC, apparent diffusion coefficient; IQR, interquartile range; kPA, kilopascal; HER2, Human Epidermal Growth Factor 2; Luminal A, tumors with ER positive or PR positive/HER2 negative with low Ki-67 expression; Luminal B, tumors with ER positive or PR positive/HER2 positive or HER2 negative with high Ki-67 expression; HER2 positive, tumors with ER negative and PR negative/HER2 positive; Triple-negative, tumors with ER negative, PR negative and HER2 negative.

**Table 5:** Median ADC value and elasticity according to lesion morphology.

Lesion Morphology	ADC (Median ± [IQR], ×10^−3^ cm^2^/s)	Elasticity (Median ± [IQR], kPa)	*p* Value
Malignant Lesions			
Mass (*n* = 117)	0.94 (0.25)	126.00 (27.50)	ADC → 0.444 Elasticity → 0.718
Non-mass enhancement (*n* = 17)	0.93 (0.28)	135.00 (22.00)	
Benign Lesions			
Mass (*n* = 6)	1.60 (0.67)	30.50 (94.75)	ADC → 0.836 Elasticity → 0.628
Non-mass enhancement (*n* = 7)	1.57 (0.81)	64.50 (129.00)	

ADC, apparent diffusion coefficient; IQR, interquartile range; kPA, kilopascal.

**Table 6:** Comparison between previous studies and our study (A) for elasticity and (B) for DWI.

**ELASTICITY**							
	**Year of Publication**	**Number of Malignant Lesions**	**ER and PR Positivity Cut-Off Value**	**Ki-67 Proliferation Index Cut-Off Value**	**HER2 Positivity Assessment Method**	**Elastography Type**	**Findings**
Youk et al. [17]	2013	166	Method of Quick (Allred) Score (QS)	≥14%	Score 3 or 2 and HER2 amplification	SWE	- ER, PR, Ki-67 expressions and lesions size were associated with elasticity - HER2 was not associated with elasticity - Among subtypes, only TNBC showed significant association with elasticity
Chang et al. [18]	2013	337	≥10%	Not mentioned	Score 3 or 2 and HER2 amplification	SWE	- Lesion size, ER, PR and molecular subtypes were associated with elasticity
Choi et al. [19]	2014	122	Not mentioned	Not mentioned	Not mentioned	SWE	- Lesion size, ER, PR and Ki-67 were associated with elasticity - Molecular subtypes were not associated with elasticity
Ganau et al. [20]	2015	216	≥10%	≥14%	Score 3 or 2 and HER2 amplification	SWE	- Lesion size was significantly correlated with elasticity - ER, PR, Ki-67 expressions were not associated with elasticity - There was no significant difference among molecular subtypes in elasticity
Makal et al. [21]	2021	112 lesions (full malign)	≥1%	≥14%	Score 3 or 2 and HER2 amplification	SWE	- Lesion size was associated with elasticity - ER, PR, HER2 and Ki-67 were not associated with elasticity - Among subtypes; there was no correlation with elasticity
Our study		133	≥1%	≥14%	Score 3 or 2 and HER2 amplification	SWE	- Lesion size, ER, PR, HER2 and Ki-67 index were not associated with elasticity - Among subtypes; there was no correlation with elasticity
**ADC**							
	**Year of Publication**	**Number of Malignant Lesions**	**ER and PR positivity cut-off value**	**Ki-67 proliferation index cut-off value**	**HER2 positivity assessment method**	**b Value**	**Findings**
Sung et al. [22]	2009	62	≥10%	Not mentioned	Score 2 and 3	0 and 1000	- Lesion size, ER, PR, HER2 and Ki-67 index were not associated with ADC values
Jeh et al. [23]	2011	107	≥10%	≥15%	Score 2 and 3	0, 750 and 1000	- ER and HER2 were associated with ADC values - Lesion size, PR and Ki-67 were not associated with ADC values
Choi et al. [24]	2012	335	≥10%	≥20%	Score 2 and 3	0 and 1000	- ER, PR and Ki-67 were associated with ADC values - Lesion size and - HER2 were not associated with ADC values
Moutinho-Guilherme et al. [25]	2018	100	Method of Quick (Allred) Score (QS)	≥20%	Score 3 or 2 and HER2 amplification	0 and 700	- ER was correlated with ADC values - Lesion size, PR, HER2 and Ki-67 were not associated with ADC values.
Tezcan et al. [26]	2019	83	≥10%	Not mentioned	Score 3 or 2 and HER2 amplification	0 and 500	- ER and HER2 were not associated with ADC values. - PR was associated with ADC values
Ren et al. [27]	2019	307	≥10%	≥14%	Score 3 or 2 and HER2 amplification	0 and 1000	- ER, PR, HER2 and Ki-67 were associated with ADC values
Surov et al. [65]	2019	661	Not mentioned	≥14%	Not mentioned	Different b values	- Among subtypes; there was no correlation with ADC values - Ki-67 index was weakly correlated with ADC values
Linh et al. [29]	2021	49	≥1%	≥14%	Score 3 or 2 and HER2 amplification	0 and 1000	- ER, PR, HER2 and molecular subtypes were not associated with ADC values - Ki-67 index was correlated with ADC values
Our study		133	≥1%	≥14%	Score 3 or 2 and HER2 amplification	0 and 600	- Lesion size, ER, PR, HER2 and Ki-67 index were not associated with ADC - Among subtypes; there was no correlation with ADC

DWI; diffusion-weighted imaging; ER, estrogen; PR, progesterone; Ki-67, Nuclear protein Ki-67; HER2, Human Epidermal Growth Factor 2; TNBC, triple-negative breast cancer; SWE, shear-wave elastography; ADC, apparent diffusion coefficient.

## Data Availability

Not applicable.
